# Valproic acid attenuates the severity of astrogliosis in the hippocampus of animal models of temporal lobe epilepsy

**DOI:** 10.1016/j.ibneur.2024.11.003

**Published:** 2024-11-08

**Authors:** Hu Feng, Jiamin Luo, Zhiwei Li, Yuxiao Zhao, Yamei Liu, Hongyan Zhu

**Affiliations:** School of Life Sciences, Shanghai University, Nanchen Road 333, Shanghai 200436, China

**Keywords:** Valproic acid, Reactive astrogliosis, Neuronal loss, Temporal lobe epilepsy

## Abstract

Reactive astrogliosis is one of the most frequency neuropathological alterations in the hippocampus of animal models and patients with temporal lobe epilepsy (TLE). Valproic acid (VPA), a widely used antiepileptic drug (AED), acts by blocking ion channels and enhancing GABAergic activity. This study investigated the effects of VPA on hippocampal astrogliosis in a rat model of TLE. The results demonstrated that chronic administration of VPA at a dose of 200 mg/kg significantly reduced the severity of astrogliosis and ameliorated neuronal loss in the hippocampus at the early and middle stages post-status epilepticus (SE), while also improving cognitive impairments at the middle and late stages in KA-SE rats. Long-term administration of VPA at 400 mg/kg attenuated astrogliosis in the hippocampus at the middle stage post-SE, but lacked neuroprotective effects and exacerbated cognitive impairments at the late stage. These findings suggest that VPA at an appropriate dose could mitigate hippocampal astrogliosis, potentially offering a new antiepileptic mechanism for its long-term use.

## 1 Introduction

Temporal lobe epilepsy (TLE) is a group of neurological disorders characterized by recurrent, unprovoked seizures arising from one or both temporal lobes of the brain (Becker, 2018). Hippocampal sclerosis (HS) is considered the most common pathological feature of TLE, and is characterized by the selective loss of pyramidal neurons in the CA1 and CA3 areas and reactive gliosis in the hippocampus (Bianchin et al., 2014, Blumcke et al., 2013, Bruxel et al., 2021, Sendrowski and Sobaniec, 2013). The seizure-related neuronal loss of the hippocampus is regarded to generally results from excitotoxic glutamatergic neurotransmission and excessive Na^+^ and Ca^2+^ influx (Thom, 2014). Reactive astrogliosis, another prominent feature of HS, is referred to as a process that astrocytes respond to all forms of CNS insults, in which a finely gradated continuum of changes occur in context-dependent manners and range from reversible alterations in gene expression, and hypertrophy of cell bodies and processes. (Escartin et al., 2019, Jakel and Dimou, 2017, Patel et al., 2019). A growing body of evidence revealed that the dysfunction of astrocytes in epileptic brain may be involved in the epileptogenesis via disturbed energy metabolism and water homeostasis, the alterations in extracellular ion, and the abnormal release of neurotransmitter such as glutamate (Binder and Steinhauser, 2021, Boison and Steinhauser, 2018, Heuser and Enger, 2021, Patel et al., 2019, Steinhauser et al., 2016).

Previous work in our lab demonstrated that Nav1.6, the most abundant subtype of voltage-gated sodium channels (VGSCs) in the adult mammalian brain, is highly expressed in reactive astrocytes of the hippocampus in a post-SE animal model of TLE. The expression of Nav1.6 was strongly correlated with the severity of reactive astrogliosis, suggesting that increased VGSC expression is a significant molecular alteration in the process of reactive astrogliosis (Zhu et al., 2016). Additionally, Nav1.5, another VGSCs subtype, has been implicated in astrogliosis in an in vitro model of glial injury (Pappalardo et al., 2014), raising the possibility that VGSCs contribute to reactive astrogliosis. Valproic acid (VPA), a commonly used antiepileptic drug, exerts its effects by elevating GABA levels, reducing aspartate levels, and blocking sodium, calcium, and potassium channels, including VGSCs, voltage-gated calcium channels, and voltage-gated potassium channels (Chateauvieux et al., 2010, Taverna et al., 1998). Furthermore, VPA is a potent epigenetic agent, inhibiting histone deacetylases (HDACs), affecting DNA and histone methylation, and altering the expression of transcription factors to regulate gene expression (Mello, 2021, Romoli et al., 2019). Specifically, VPA has been shown to downregulate the expression of SCN3A, a VGSC subtype, in mouse Neuro-2a cells and reduce Nav1.3 expression in the hippocampus of seizure models (Tan et al., 2017). Given the correlation between increased VGSC expression and reactive astrocytes, and the role of VPA as a VGSC blocker and epigenetic agent, we hypothesize that chronic VPA treatment may influence reactive astrogliosis.

In this, a post-SE animal model of TLE was induced by kainic acid (KA) intrahippocampal injection. We then investigated the effects of long-term VPA administration on hippocampal astrogliosis and neuronal damage, and cognition at the latent and chronic stages after SE in KA-SE rats.

## 2 Materials and methods

### 2.1 Animal

Male Sprague Dawley rats weighing 210–240 g were purchased from Shanghai experimental animal center (Chinese Academy of Sciences). The rats were kept in individual cages with food and water ad libitum under certain environment (21–25 °C; 12 h light/dark cycle). The experimental procedures were approved by the Committee of Laboratory Animals, Shanghai University.

### 2.2 KA induced post status epilepticus model

KA-induced post status epilepticus (SE) model was established in accordance with the protocol described in our previous report (Zhu et al., 2016). A gas mixture comprising 3 % isoflurane was used to anesthetize the rats. Then the rats were placed in a stereotaxic device (Narishige, Tokyo, Japan). KA (0.5 μg in 2 μl saline, at the rate of 0.5 μl/min) was unilaterally injected into the CA3 region of the right hippocampus according to the coordinates of Paxinos atlas (2004): AP = - 4.1 mm, ML= - 4.2 mm, DV= - 4.3 mm. The same volume of saline was injected into the same place for controls. Rats were monitored by a video recording. Racine’s scale (stage 1–5 seizures) was used to score seizure activities. SE was defined by continuous motor seizure activities corresponding several stage 4 seizures or at least one stage 5 seizure for at least 2 h (Mazarati et al., 2008, Puttachary et al., 2016, Zhu et al., 2016). Those animals that failed to develop stage 4–5 seizures were excluded from further experiments. For increasing the survival of rats with SE, diazepam (5 mg/kg) was by intraperitoneal injection after 4 h of SE. After recovering from SE, spontaneous seizures (SRSs) were continuously monitored for 12 h each day in each KA-SE rat by a video-monitoring system from week 1 to 9. The criterion of SRS is defined by motor seizure activities corresponding to the stages from 3 to 5 according to the scale of Racine.

### 2.3 Chronic treatment with VPA

**Fig. 1:**
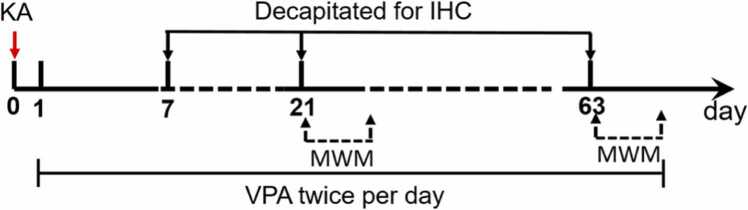
The timeline for the experimental design. IHC: immunohistochemistry; MWM: Morris water maze.

At 1 day after KA injection, these KA-SE rats were randomly divided into three groups, so that the severity of SE did not significantly differ between the groups. Two groups were treated with VPA (Sigma Aldrich GmbH) diluted in saline at the dose of 200 mg/kg, 400 m g/kg, saline, twice per day (at 8 a.m. and 8 p.m.), respectively. The third group received saline injections. The dose of VPA was set according to the previous studies (Brandt et al., 2006, Hassel et al., 2001). There is no obvious difference in the severity of SE among the groups. The sham rats were also randomly divided into three groups treated with saline, VPA at the dose of 200 mg/kg, 400 m g/kg, saline, twice per day (at 8 a.m. and 8 p.m.), respectively. For immunohistochemistry, animals were decapitated for at 7, 21 and 63 days post SE. Morris water maze tests were performed at 21 and 63 days after SE, respectively. The experimental design is shown in Fig. 1.

### 2.4 Immunohistochemistry

Rats were deeply anesthetized with 2 % sodium pentobarbital and then transcardially perfused with 4 % paraformaldehyde (PFA) in 0.1 M PB (pH = 7.4). Brains were removed and postfixed for 24 h in 4 % PFA at 4 °C and then stored in 20 % and 30 % sucrose in 0.1 M PB for cryoprotection. A series of coronal sections with 20 μm-thick were cut on a freezing microtome (Leica, Germany). The brain sections were washed 3 times with 0.01 M PBS (pH = 7.4) for 5 min each, then incubated in 0.5 % Triton X-100 containing 3 % H_2_O_2_ for 30 min. Next, the sections were washed 3 times with 0.01 M PBS for 5 min each, then incubated in blocking serum (5 % normal goat serum). The sections were incubated with the primary antibodies including a monoclonal mouse anti-NeuN antibody (1:500, Cat.No: #104224, Abcam) and a monoclonal mouse anti- Glial fibrillary acid protein (GFAP) (1:300, Cat. No: ab190288, Abcam) diluted in 5 % normal goat serum containing 0.5 % Triton X-100 overnight at 4 °C. On the secondary day, the sections were incubated with biotinylated goat anti-mouse secondary antibody (1:200, Cat.No: #8310-08, Southern Biotech) for 1 h. Next, the sections were washed 3 times with 0.01 M PBS for 5 min each, then incubated with the avidin-biotin complex (ABC) solution (1:200 in 0.01 M PBS containing 5 % goat serum, ABC-Elite kit, Vector Laboratories) for 1 h, washed with 0.01 M PBS again, and incubated with DAB substrate for 10 min in darkness. Finally, the reaction was visualized with 3,3´-diaminobenzidine tetrahydrochloride (DAB) with 0.01 % H_2_O_2_ in 0.01 M PBS.

### 2.5 Morris water maze

Morris water maze (MWM) tests were carried out to evaluate spatial learning and memory in KA-SE rats. Rats used in Morris water maze test were divided into two cohorts, which are tested at 3 weeks and 9 weeks after SE, respectively. Each cohort was divided into four groups: sham rats, KA-SE rats treated with saline, KA-SE rats treated with VPA at dose of 200 mg, KA-SE rats treated with VPA at dose of 400 mg sham. There are 6–8 rats each group. The tests were carried out as following: The rats were place into a circular fiberglass pool (200 cm diameter) filled with water (25 °C ± 1 °C) containing nontoxic black paint to freely swim for two days for the habituation. A hidden platform was submerged 2.4 cm below the water surface and located in the center of one of four quadrants. The freely swimming rats can learn to escape from water onto the platform. In order to record the swim paths of the animals, a video-monitoring system hung above the pool center was used. On the next 4 consecutive days, the rats were placed into the water maze from four different starting points at four quadrants to swim until they found the hidden platform for 4 trials per day. If the rats did not detect the hidden platform within 60 s, they were guided to the platform. MWM tests were performed in the rats at 21 and 63 days after KA injection.

### 2.6 Tissue analysis

Images were captured with a Nikon microscope operating and saved as 16-bit TIFF files. Images from the identical regions and layers of the hippocampus in VPA- and saline-treated rats (5–6 animals for each group) were processed in parallel. The relative optical density of the immunopositive cells was assessed by the Image J software (Wayne Rasband, National Institute of Health, USA). Briefly, the mean optical density (MOD) of GFAP- immunoreactivity (IR) was assessed in two 400 μm × 250 μm grids, and the MOD of NeuN-IR were assessed in two 400 μm × 150 μm grids. These grids were laid over the hippocampal subareas of CA1, CA3 and hilus in six coronal sections (from – 2.64 mm to – 3.96 mm from Bregma) each rat, respectively. RGB (24-bits) color images were converted to 8-bit grayscale images (0–255 gray levels). Blood vessels and other artifacts were avoided and the background correction was performed according to the formula previously described (Xavier et al., 2005). The analysis was carried out by an experimenter blind to the experimental procedures.

### 2.7 Statistics analysis

All data were expressed as mean ± S.E.M in the study. One-Way ANOVA followed by a post hoc Turkey test was carried out to assess the significant differences among the groups. In MWM tests, the differences were analyzed by repeated measures ANOVA with group and training day as sources of variance. The statistical analyses were performed by SPSS 22.0. All data were analyzed by the one who was unaware of the experimental procedures.

## 3 Results

### 3.1 Spontaneous seizures alterations in KA-SE rats during treatment with vehicle or VPA

**Fig. 2:**
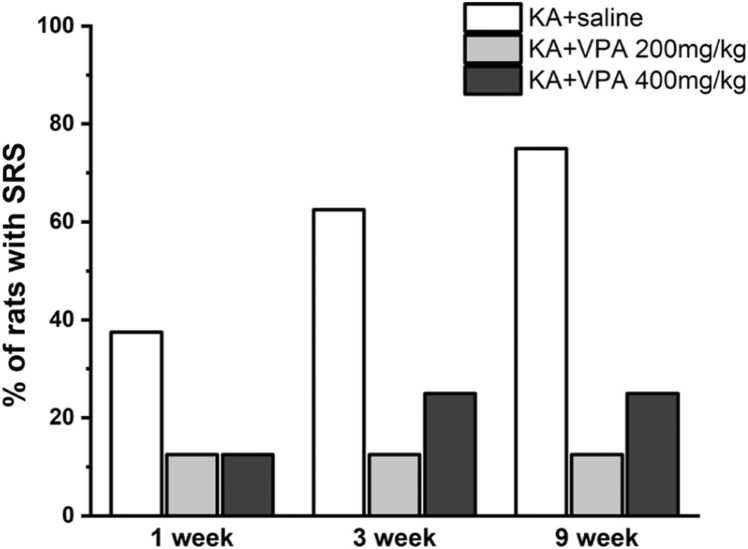
Recording for SRS following the onset of SE in rats treated with VPA or saline at different time points after SE. The numbers on the columns present the number of rats with SRS each group. n = 8 rats per group.

KA (0.5 μg) was injected into the CA3 subarea of the right dorsal hippocampus of rats to induce status epilepticus (SE). After 2 h of SE, KA-SE rats were received the first intraperitoneal injection of saline or VPA. Spontaneous recurrent seizures (SRS) were collected and analyzed at 1 week, 3 weeks and 9 weeks after SE in KA-SE rats with the treatment of saline or VPA. Fig. 2 showed that the percentage of rats with SRS was remarkably reduced in KA-SE rats treated with VPA compared to KA-SE rats treated with saline. However, the long-term administration of VPA cannot prevent the onset of SRS in KA-SE rats after SE.

### 3.2 Reduction of the astrogliosis in the ipsilateral hippocampus of KA-SE rats treated with VPA

**Fig. 3:**
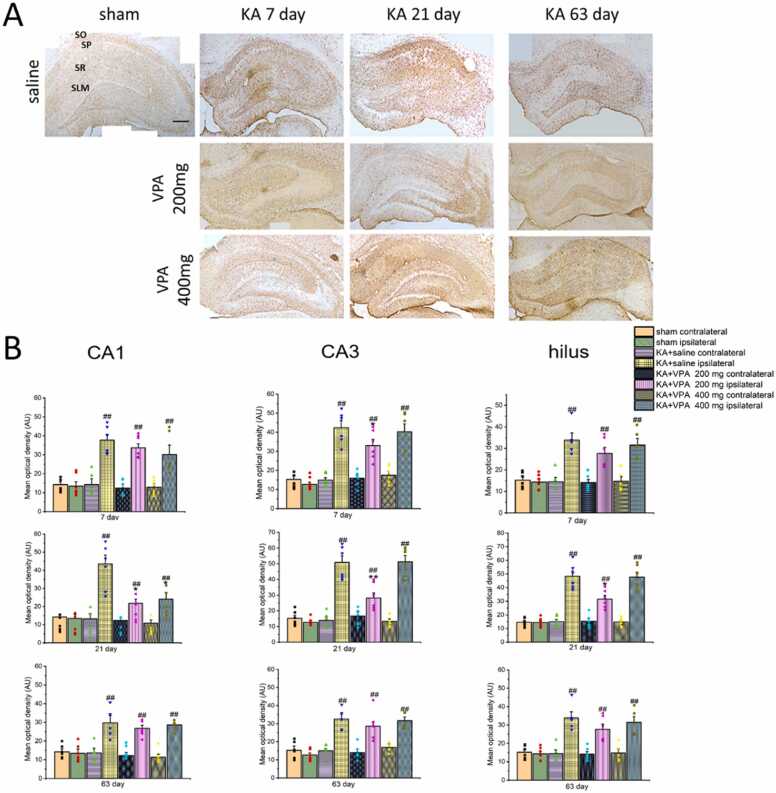
VPA alleviates GFAP-IR in the ipsilateral hippocampus of KA -SE rats. A: Representative photographs of GFAP-IR in the ipsilateral hippocampus in the rats treated with saline and VPA. B: Histograms demonstrate the mean optical density of GFAP-IR in CA1 CA3, and hilus subareas of the ipsilateral hippocampus in shams and KA-SE rats treated with saline and VPA at dose of 200 and 400 mg/kg at the different time points post SE. * p < 0.05, ** p < 0.01, significantly different from the ipsilateral hippocampus in the KA-SE rats treated with saline. # p < 0.05, ## p < 0.01, significantly different from the ipsilateral hippocampus in the sham rats. SO: stratum oriens; SP: stratum pyramidale; SR: stratum radiatum; n= 5–6 rats per group. Scale bar = 300 μm.

**Fig. 4:**
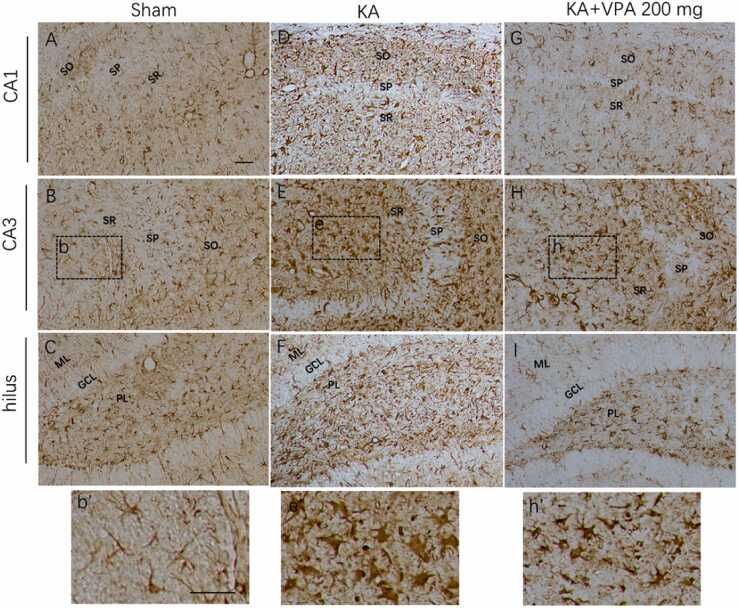
VPA at dose of 200 mg/kg reduces reactive astrogliosis in the ipsilateral hippocampus of KA-SE rats at 21 days post-SE. Compared to the sham rats (A–C), KA-SE rats exhibit increased GFAP-IR and the hypertrophy of bodies and processes in astrocytes of the CA1, CA3 and hilus in the ipsilateral hippocampus (D–F). VPA at dose of 200 mg/kg decreases GFAP-IR and the hypertrophy of astrocytes in the CA1, CA3 and hilus of the ipsilateral hippocampus in KA-SE rats (G–I). The areas in dashed box of b, e and h are magnified in b’, e’ and h’. SO: stratum oriens; SP: stratum pyramidale; SR: stratum radiatum; ML: molecular layer; GCL: granule cell layer; PL, polymorphic layer; Scale bar = 30 μm.

Reactive astrogliosis in the hippocampus was evaluated by Glial fibrillary acid protein (GFAP) immunoreactivity, a prototypical marker for astrocytes, by immunostaining. As shown in Fig. 3 A, GFAP-IR was remarkably augmented in the whole ipsilateral hippocampus of KA-SE rats treated with saline at 7, 21 and 63 days post-SE. The MOD of GFAP-IR was significantly increased in the CA1, CA3 and hilus of ipsilateral hippocampus at 7, 21 and 63 days post-SE (p < 0.01, respectively) (Fig. 3 B). Notably, the MOD of GFAP-IR in the ipsilateral hippocampus at 63 days was significantly decreased compared with that of 7 and 21 day post-SE (p < 0.01, respectively). Furthermore, the most severity of reactive astrogliosis was identified in the CA3 subarea of ipsilateral hippocampus at 21 days post-SE, exhibiting the hypertrophy of cell bodies and processes, pronounced overlap of processes as well as cell proliferation (Fig. 4). Although reactive astrogliosis was also found in the ipsilateral hippocampus of KA-SE rats treated with VPA (at dose of 200 and 400 mg/kg, respectively), the severity of reactive astrogliosis was remarkably reduced in the ipsilateral hippocampus of KA-SE rats with the long term treatment of VPA compared to KA-SE rats treated with saline. Specifically, GFAP-IR was significantly decreased in the ipsilateral CA3 at 7 days, the ipsilateral CA1, CA3 and hilus at 21 days post-SE, in KA-SE rats treated with VPA at dose of 200 mg/kg compared to the saline-treated group (Fig. 3 B; p < 0.05, p < 0.01, respectively). GFAP-IR was significantly decreased in the ipsilateral CA3 at 21 days post-SE in KA-SE rats treated with VPA at dose of 400 mg/kg compared to the saline-treated group (Fig. 4 3 B; p < 0.05). However, VPA at dose of 200 mg/kg or 400 mg/kg had no influences on GFAP-IR in the ipsilateral hippocampus at 63 days post-SE (Fig. 3 A and B) (p > 0.05, respectively). Furthermore, the hypertrophy of astrocyte in the ipsilateral hippocampus was prominently reduced by the long term treatment of VPA at 200 and 400 mg of KA-SE rats at 7 and 21 days post-SE (Fig. 4). Moreover, VPA at 200 or 400 mg had no influence on GFAP-IR in the ipsilateral hippocampus in sham rats (p > 0.05, respectively).

### 3.3 Neuroprotective effects of VPA on the ipsilateral hippocampus in KA-SE rats

**Fig. 5:**
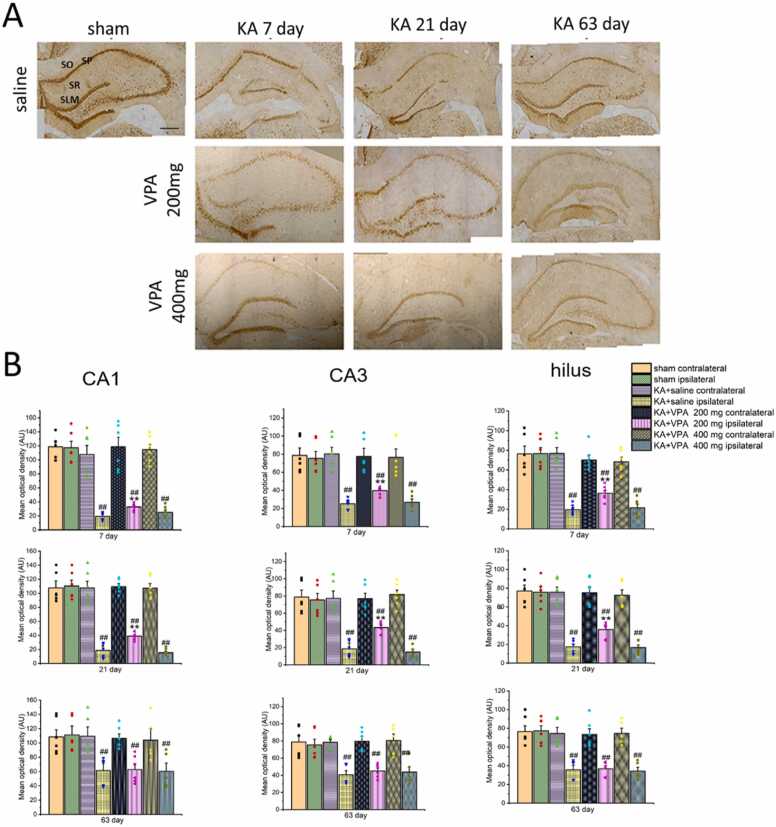
VPA has neuroprotective effects on the ipsilateral hippocampus of KA-SE rats. A: Representative photomicrographs of NeuN-IR in the ipsilateral hippocampus in the sham and KA-SE rats treated with saline and VPA at 7, 21 and 63 days post-SE. B: Histograms show that VAP can significantly increase the MOD of NeuN-IR in the CA1, CA3 and hilus subareas of ipsilateral hippocampus in KA-SE rats * p < 0.05, ** p < 0.01, significantly different from the ipsilateral hippocampus of KA-SE rats treated with saline. # p < 0.05, ## p < 0.01, significantly different from the ipsilateral hippocampus in the sham rats. SO: stratum oriens; SP: stratum pyramidale; SR: stratum radiatum; ML: molecular layer; GCL: granule cell layer; PL, polymorphic layer; Scale bar = 300 μm; n= 6 rats per group.

**Fig. 6:**
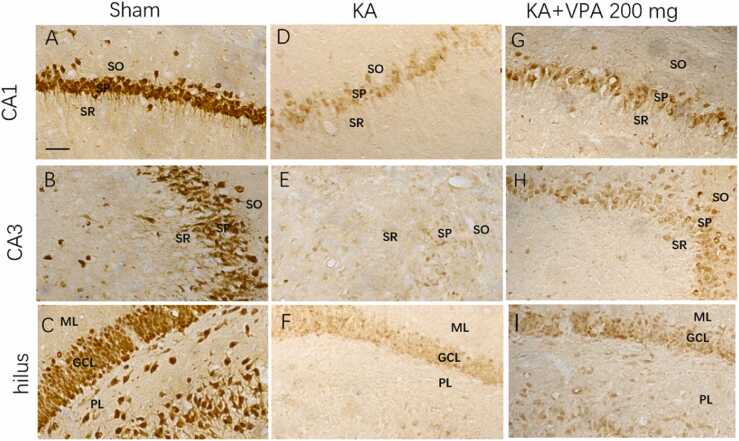
VPA at dose of 200 mg/kg attenuates neuronal loss in the ipsilateral hippocampus of KA-SE rats at 21 days post-SE. NeuN-IR positive neurons are prominently decreased in the CA1, CA3 and hilus of ipsilateral hippocampus in KA-SE (D–F) compared to the sham rats (A–C), and VPA at dose of 200 mg/kg can rescue neuronal loss of the ipsilateral hippocampus in KA-SE rats at 21 days post-SE (G–I). The areas in dashed box of b, e and h are magnified in b’, e’ and h’. SO: stratum oriens; SP: stratum pyramidale; SR: stratum radiatum; ML: molecular layer; GCL: granule cell layer; PL, polymorphic layer; Scale bar = 50 μm.

NeuN-immunostaining showed severe neuronal loss in the ipsilateral hippocampus of KA-SE rats treated with saline at 7, 21 and 63 days after SE compared to sham rats (Fig. 5). The most severe neuron loss was observed in the ipsilateral hippocampus in KA-SE rats at 21 days post-SE (Fig. 5, Fig. 6). Notably, NeuN-IR at 63 day post-SE was significantly increased in the CA1, CA3 and hilus of ipsilateral hippocampus compared to that of the ipsilateral hippocampus at 7 and 21 day after SE, (p < 0.01, respectively) (Fig. 5). The administration of VPA at dose of 200 mg/kg can significantly improve the neuronal loss in the CA1, CA3 and hilus of ipsilateral hippocampus of KA-SE rats at 7 and 21 days after SE (p < 0.01, respectively) (Fig. 5, Fig. 6). Interestingly, we did not observe the neuroprotective effects of VPA at dose of 400 mg/kg on the CA1, CA3 and hilus of ipsilateral hippocampus in KA-SE rats at 7 and 21 days after SE (p > 0.05, respectively) (Fig. 5). VPA at dose of 200 mg/kg or 400 mg/kg has no effects on the neuron damages of the ipsilateral hippocampus in KA-SE rats at 63 days after SE (p > 0.05, respectively) (Fig. 5). Moreover, VPA at 200 or 400 mg had no influence on NeuN-IR in the ipsilateral hippocampus in sham rats (p > 0.05, respectively).

### 3.4 Improvements of cognitive impairments in KA-SE rats treated with VPA

**Fig. 7:**
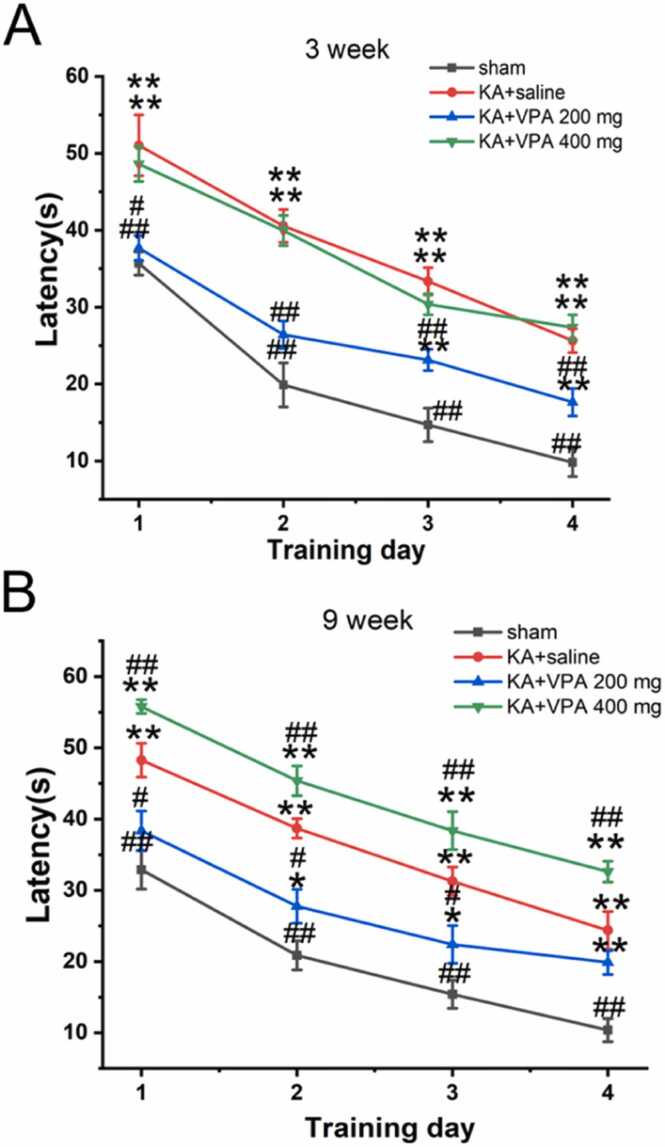
Acquisition learning of platform location by KA-SE rats treated with saline and VPA in Morris water maze. The latency in the figure represents the mean time that the rats reach the hidden platform during the 4 days of water maze testing. * p < 0.05, ** p < 0.01, significantly different from the rats treated with saline in the same trial. # p < 0.05, ## p < 0.01, significantly different from the ipsilateral hippocampus in the sham rats in the same trial. n = 6–8 rats per group.

To evaluate the influences of VPA on the hippocampal function in KA-SE rats, Morris water maze (MWM) test was carried out. In MWM test, the latency to find the hidden platform was measured. The saline-treated KA-SE rats showed the longer latency at 3 weeks and 9 weeks after SE compared to the sham rats (3w: F_(1, 15)_ = 24.4, p < 0.01; 9 w: F_(1, 15)_ = 57.3, p < 0.01) (Fig. 7). The latency to locate the hidden platform of KA-SE rats treated with low-dose VPA (200 mg/kg) was shorter than KA-SE rats treated with saline at 3 weeks and 9 weeks after SE (p < 0.01) (3w: F_(1, 15)_ = 9.212, p < 0.01; 9 w: F_(1, 15)_ = 31.64, p < 0.01). However, the effects of high-dose VPA (400 mg/kg) on the latency are remarkably differed from low-dose VPA (200 mg/kg) in KA-SE rats at 3 weeks and 9 weeks after SE. There is no difference of the latency to find the hidden platform between KA-SE rats treated with VPA at dose of 400 mg/kg and KA-SE rats treated with saline at 3 weeks after SE (F_(1, 15)_ = 0.082, p > 0.05). Furthermore, it took KA-SE rats treated with VPA at dose of 400 mg/kg longer time to find the hidden platform than KA-SE rats treated with saline at 9 weeks after SE (F_(1, 15)_ = 7.74, p < 0.05). The above results suggested that VPA at low dose effectively improve cognitive deficits, but VPA at high dose exacerbate cognitive impairments in epileptic rats.

## 4 Discussion

The present study reveals that long-term administration of VPA has the anti-astrogliosis effects on the ipsilateral hippocampus of KA-SE rats. Specifically, long-term administration of VPA at an appropriate dose reduces GFAP-IR and the hypertrophy of reactive astrocytes in the ipsilateral hippocampus in KA-SE rats at 7 and 21 days after SE. Furthermore, it attenuates neuronal loss and improves cognitive impairments in the latency and chronic phase of epileptogenesis in KA-SE rats. However, despite reducing astrogliosis, high-dose VPA at has no effects on neuronal loss and aggravates cognitive impairments in the chronic phase of epileptogenesis in KA-SE rats.

Reactive astrogliosis is an adaptive process in response to diverse forms of CNS insults and diseases, during which reactive astrocytes undergo a gradated continuum of alterations that vary with the type and severity of injury, including cellular hypertrophy, proliferation and molecular expression, and exhibit different cellular morphology corresponding to mild or moderate reactive astrogliosis, severe diffuse reactive astrogliosis, severe diffuse reactive astrogliosis with compact scar (Binder and Steinhauser, 2021, Seifert and Steinhauser, 2013, Sofroniew, 2005, Sofroniew and Vinters, 2010). Several studies demonstrated reactive astrogliosis present in most animal models of acquired epilepsy and patients with epilepsy, and suggested the involvement of reactive astrogliosis in epileptogenesis (Ortinski et al., 2010, Patel et al., 2019, Robel et al., 2015). For example, the conditional deletion of astrocyte- specific β1 integrin gene induce widespread chronic astrogliosis in mice which develop spontaneous and recurrent seizures (Robel et al., 2015). The selective induction of astrogliosis in the hippocampus generates rapid synaptic GABA depletion and cause neuronal hyperexcitability (Ortinski et al., 2010). Consistent with previous studies, the present research reveals the occurrence of reactive astrogliosis throughout the ipsilateral hippocampus in KA-SE rats at 7, 21, and 63 days post-SE. The most severe astrogliosis was observed at 21 days post-SE, characterized by astrocyte hypertrophy, proliferation, and pronounced overlap of processes. Continuous VPA treatment at 200 and 400 mg/kg significantly reduced astrocyte hypertrophy in the ipsilateral hippocampus during the latent stage. Moreover, the anti-astrogliosis effect of VPA varied by dose; 200 mg/kg was more effective than 400 mg/kg during the latent stage. However, neither dose impacted astrogliosis in the chronic stage. These findings suggest that the effects of VPA on astrogliosis are dose-dependent and influenced by the severity of the condition. A study by Brandt et al. (2006) reported a reduction in glial cells in the hilus of the hippocampus in epileptic rats during the chronic stage, using thionin staining. This discrepancy may stem from differences in dosage and treatment regimens. Intracellular signaling pathways such as STAT3, Nrf2, cAMP et al. have been proposed to mediate reactive astrogliosis, including astrocyte hypertrophy and proliferation (Sofroniew, 2009). Given that VPA has epigenetic effects by inducing histone acetylation, affecting DNA and histone methylation status, and altering gene expression (Mello, 2021, Romoli et al., 2019), the reduction of reactive astrogliosis in KA-SE rats may be attributed to VPA's modulation of gene expression involved in astrogliosis. Additionally, Pappalardo et al. demonstrated that Nav1.5 contributes to astrogliosis in an in vitro model of glial injury (Pappalardo et al., 2014). Our unpublished data show that downregulation of Nav1.6 reduces astrogliosis in the hippocampus of KA-SE rats. The blockade of VGSCs may play a key role in the ant-astrogliosis effects of VPA.

Although long-term administration of VPA cannot prevent the onset of SRS in KA-SE rats following SE, it significantly reduces SRS in these animals. This finding aligns with Brandt et al. (2006), who demonstrated that chronic VPA treatment exerted neuroprotective effects against neuronal damage in the hippocampus after SE but failed to prevent the development of epileptogenesis in post-SE TLE animals. In epileptic brain, accompanying the hypertrophy, reactive astrocytes exhibit physiological and molecular alterations, including impairment in ion and neurotransmitter homeostasis, the changed expression in enzymes, transporters and channels such as glutamine synthetase, adenosine kinase, KCC2, EAAT1, EAAT2, Kir4.1, AQP4 et al. that are proposed to contribute to neuronal hyperexcitability in KA-SE rats (Devinsky et al., 2013, Patel et al., 2019). The reduction in SRS by VPA may partially be attributed to its anti-astrogliosis effects, which mitigate the epileptic hyperexcitability caused by astrogliosis.

Severe neuronal loss was observed in the ipsilateral hippocampus of KA-SE rats in our present experiments, which is consistent with previous reported (Bianchin et al., 2014, Blumcke et al., 2013, Brandt et al., 2006, Bruxel et al., 2021, Sendrowski and Sobaniec, 2013). Moreover, several lines of evidence suggested that neuronal loss following SE is associated with cognitive impairments in temporal lobe epilepsy (Tai et al., 2018). Neuronal loss in the ipsilateral hippocampus and spatial cognitive impairments were indeed observed in KA-SE rats in the present experiments. Continuous administration of 200 mg/kg VPA significantly mitigated neuronal loss in the ipsilateral hippocampus during the latent stage and alleviated cognitive impairments in both the latent and chronic stages in KA-SE rats. Previous studies have demonstrated that chronic VPA treatment prevents neuronal damage (Chateauvieux et al., 2010). A growing evidence have suggested that the neuroprotective effects of long-term VPA treatment may be attributed to its epigenetic regulation of genes related to synaptic receptors and several signaling molecules involved in excitotoxic and neuroprotective pathways, such as the serotonin-2A (5-HT2A) receptor, GAP-43, brain-derived neurotrophic factor (BDNF), glial cell-derived neurotrophic factor (GDNF), and SCN3 (Romoli et al., 2019). On the other hand, reactive astrogliosis may have detrimental effects by exacerbating inflammation, increasing extracellular glutamate, and impairing GABAergic inhibition, all of which contribute to excitotoxicity, excessive inflammation, and neuronal death (Steinhauser et al., 2016). The neuroprotective effects of long-term VPA administration may be partly due to its ability to counteract these processes by reducing astrogliosis in KA-SE rats.

As the metabolism of VPA is very rapid in the rats, high doses of VPA is regularly used to achieve relevant serum concentrations (40–100 mg/ML) known from patients with epilepsy (Loscher, 1999). The dose of VPA used in the experiments was set based on two studies (Brandt et al., 2006, Hassel et al., 2001). Our experiments showed that rats treated with 400 mg/kg of VPA experienced approximately 5 % weight loss by day 63, which is similar to Hassel’s study. Continuous administration of VPA at a higher dose (400 mg/kg) did not reduce neuronal loss or improve cognitive impairments during the latent stage after SE and even exacerbated cognitive impairments in the late stage in KA-SE rats. Several clinical studies have also highlighted the correlation between VPA treatment and cognitive impairment. For instance, VPA used for pain management reduced cognitive performance in HIV patients (Cysique et al., 2006). Masmoudi et al. reported reversible parkinsonism and/or cognitive impairment in epileptic patients associated with VPA treatment (Masmoudi et al., 2006). Moreover, prenatal exposure to VPA has been linked to fetal teratogenicity, intellectual disability, and an increased risk of autism (Schneider and Przewlocki, 2005). Chronic VPA treatment in male mice has also been shown to cause behavioral changes in offspring, including alterations in light/dark transition tests (Sakai et al., 2023). Additionally, hippocampal neurons in the offspring of VPA-treated mice exhibited abnormal morphology and activity (Juliandi et al., 2015). On the other hand, the drug regimen may influence the neuroprotective effects of VPA. Continuous intravenous infusion of VPA for 24 h immediately following SE has been shown to provide the most effective neuroprotection, preventing the majority of neuronal damage (Langer et al., 2011). These findings suggest that chronic high-dose VPA treatment may impair brain function, posing a potential risk of cognitive impairment in long-term clinical use. The side effects of VPA may be attributed to its epigenetic effects, altering DNA acetylation and methylation, hence regulating gene expression.

In conclusion, chronic treatment of VPA at proper dose reduces reactive astrogliosis and has neuroprotective effects in the ipsilateral hippocampus in KA-SE rats. The anti-astrogliosis of VPA action may be a new antiepileptic mechanism for the long-term administration of VPA at proper dose. However, the long-term administration of high-dose VPA slightly reduces the astrogliosis in the hippocampus, and has no neuroprotective effects and aggravates cognitive impairment in KA-SE rats,

## CRediT authorship contribution statement

**Yuxiao Zhao:** Methodology, Data curation. **Yamei Liu:** Data curation. **Hongyan Zhu:** Writing – review & editing, Supervision, Funding acquisition, Conceptualization. **Hu Feng:** Writing – original draft, Formal analysis, Data curation, Conceptualization. **Jiamin Luo:** Methodology, Formal analysis, Data curation. **Zhiwei Li:** Methodology, Data curation.

## Ethical approval

All experimental procedures were performed in accordance with the guides for animal experimentation and approved by the Committee of Laboratory Animals, Shanghai University (Approval no.: ECSHU 2021-056).

## Funding

This work was supported by 10.13039/501100001809National Natural Science Foundation of China (Grant no.: U1632120).

## Conflicts of Interest

None of the authors has any conflicts of interest. We confirm that we have read the Journal’s position on issues involved in ethical publication and affirm that this report is consistent with those guidelines.
